# Supplementary material for: Investigating the effect of clinical pharmacist intervention in transitions of care on drug-related hospital readmissions among the elderly: study protocol for a randomised controlled trial
Source: BMJ Open. 2020 Apr 27;10(4):e036650. doi: 10.1136/bmjopen-2019-036650 (PMC7213854; doi:10.1136/bmjopen-2019-036650)
Supplement: Author's manuscript [file bmjopen-2019-036650.draft_revisions.pdf]

BMJ Open is committed to open peer review. As part of this commitment we make the peer review history of every article we publish publicly available.

When an article is published we post the peer reviewers' comments and the authors' responses online. We also post the versions of the paper that were used during peer review. These are the versions that the peer review comments apply to.

The versions of the paper that follow are the versions that were submitted during the peer review process. They are not the versions of record or the final published versions. They should not be cited or distributed as the published version of this manuscript.

BMJ Open is an open access journal and the full, final, typeset and author-corrected version of record of the manuscript is available on our site with no access controls, subscription charges or pay-per-view fees (http://bmjopen.bmj.com).

If you have any questions on BMJ Open's open peer review process please email info.bmjopen@bmj.com

BMJ Open

STUDY PROTOCOL FOR A RANDOMISED CONTROLLED TRIAL INVESTIGATING THE EFFECT OF CLINICAL PHARMACIST INTERVENTION IN TRANSITIONS OF CARE ON DRUG-RELATED HOSPITAL READMISSIONS AMONG THE ELDERLY

Journal:	*BMJ Open*
Manuscript ID	bmjopen-2019-036650
Article Type:	Protocol
Date Submitted by the Author:	24-Dec-2019

Complete List of Authors:	Kindstedt, Jonas; Umeå University, Department of Integrative Medical Biology<br>Svahn, Sofia; Umeå University , Department of Integrative Medical Biology<br>Sjölander, Maria; Umeå University , Department of Integrative Medical Biology<br>Glader, E-L; Umeå University, Department of Public health and Clinical Medicine<br>Lövheim, Hugo; Umeå University , Department of Community Medicine and Rehabilitation<br>Gustafsson, Maria; Umeå University, Department of Integrative Medical Biology

Keywords:	GERIATRIC MEDICINE, CLINICAL PHARMACOLOGY, Clinical trials < THERAPEUTICS

SCHOLARONE™
Manuscripts

I, the Submitting Author has the right to grant and does grant on behalf of all authors of the Work (as defined in the below author licence), an exclusive licence and/or a non-exclusive licence for contributions from authors who are: i) UK Crown employees; ii) where BMJ has agreed a CC-BY licence shall apply, and/or iii) in accordance with the terms applicable for US Federal Government officers or employees acting as part of their official duties; on a worldwide, perpetual, irrevocable, royalty-free basis to BMJ Publishing Group Ltd ("BMJ") its licensees and where the relevant Journal is co-owned by BMJ to the co-owners of the Journal, to publish the Work in this journal and any other BMJ products and to exploit all rights, as set out in our licence.

The Submitting Author accepts and understands that any supply made under these terms is made by BMJ to the Submitting Author unless you are acting as an employee on behalf of your employer or a postgraduate student of an affiliated institution which is paying any applicable article publishing charge ("APC") for Open Access articles. Where the Submitting Author wishes to make the Work available on an Open Access basis (and intends to pay the relevant APC), the terms of reuse of such Open Access shall be governed by a Creative Commons licence – details of these licences and which Creative Commons licence will apply to this Work are set out in our licence referred to above.

Other than as permitted in any relevant BMJ Author's Self Archiving Policies, I confirm this Work has not been accepted for publication elsewhere, is not being considered for publication elsewhere and does not duplicate material already published. I confirm all authors consent to publication of this Work and authorise the granting of this licence.

For peer review only

Study protocol for a randomised controlled trial investigating the effect of clinical pharmacist intervention in transitions of care on drug-related hospital readmissions among the elderly

Jonas Kindstedt,[1] Sofia Svahn,[1] Maria Sjölander,[1] Eva-Lotta Glader,[2] Hugo Lövheim,[3] Maria Gustafsson[1]

Corresponding author

Jonas Kindstedt, Department of Integrative Medical Biology, Umeå University, Umeå, Sweden, SE-901 87 Umeå, Sweden. Phone: +46 70 657 24 22; E-mail: jonas.kindstedt@umu.se

Word count:

2,586 words

Strengths and limitations of this study

- One of few multifaceted clinical pharmacist interventions directed at transitions of care.
- Targets elderly people ≥75 years of age, a vulnerable group rarely addressed in clinical research.
- Conducted in a setting where medication reviews by clinical pharmacists are already implemented during routine hospital care.
- Based on the current trial status, the rates of withdrawal and exclusion appear to be high.

[1] Department of Integrative Medical Biology, Umeå University, Umeå, Sweden
[2] Department of Public health and Clinical Medicine, Umeå University, Umeå, Sweden
[3] Department of Community Medicine and Rehabilitation, Umeå University, Umeå, Sweden

For peer review only

Abstract

Introduction

Drug-related problems (DRPs) are a major cause of unplanned hospital admissions among elderly people, and transitions of care has been emphasised as a key area for improving patient safety. We have designed a complex clinical pharmacist intervention that targets people ≥75 years of age undergoing transitions of care from hospital to home and primary care. The main objective is to investigate if the intervention can reduce the risk of unplanned drug-related readmission within the first 180 days after the person is discharged from hospital.

Methods and analysis

This is a randomised, controlled, superiority trial with two parallel arms. A total of 700 people ≥75 years will be assigned to either intervention or routine care (control). The intervention is initiated within a week of the person being discharged from hospital and consists of repeated medical chart reviews and phone interviews. People in both study arms may have been the subject of a medication review during their ward stay. As the primary outcome, we will measure time until unplanned drug-related readmission within 180 days of leaving hospital and use log rank tests and Cox proportional-hazard models to analyse differences in survival data between the groups. Further investigations of subgroup effects and adjustments of the regression models will be based on heart failure and cognitive impairment as prognostic factors.

Ethics and dissemination

The study has been approved by the Regional Ethical Review Board in Umeå (registration numbers 2017-69-31M, 2018-83-32M, and 2018-254-32M) and registered at ClinicalTrials.gov (trial registration number ID NCT03671629). We intend to publish the results with open access in international peer-reviewed journals and present the findings of the trial at both national and international conferences. The trial is expected to result in more than one published article and form part of two PhD-theses.

For peer review only

Trial registration number

NCT03671629

Introduction

An ageing population with multiple morbidities and medications brings with it considerable challenges for healthcare and society in terms of adverse drug reactions and other drug-related problems (DRPs) (1). Hospital admission rates increase with age (2-4) and, although prevalence estimates differ between studies (5), it has been observed that up to 30% of unplanned hospitalisations among the elderly are caused by DRPs (6, 7), a percentage that appears to be even higher in the vulnerable subgroup of old people with major neurocognitive disorder (NCD) (8). The transition of care between different healthcare providers has been associated with a high risk of inappropriate prescribing, medication errors, and adverse drug events (9-11), and the World Health Organization (WHO) has identified transitions of care as a key issue to improve patient safety in primary healthcare (12).

According to review data, poor adherence is a major concern in developed countries given that approximately 50% of all people with medications (all age groups combined) do not follow their recommended therapy (13). The mechanisms of adherence are multifactorial and, even though there is little evidence to support old age alone as an independent predictor of non-adherent behaviour, the elderly are still at higher risk due to multiple morbid conditions and polypharmacy (14). Importantly, it has also been observed that even milder forms of cognitive impairment severely affect adherence to medication (15). In the context of transitions of care, poor adherence in the sensitive post-discharge phase has been associated with hospital readmission (16) and appears to be of particular clinical relevance among people suffering heart failure (17, 18).

The clinical pharmacist is one of many professions involved in preventing and managing DRPs. In principle, clinical pharmacy can be described as the science and practice of rational medication use and includes both medication reviews and counselling with patients as well as other health care professionals (19). Medication reviews conducted by clinical pharmacists have demonstrated a

For peer review only

positive overall effect on both the appropriateness and cost of medication (20, 21). Regarding the effect on hospital readmissions and other clinical outcomes, review data are less consistent and incorporate few randomised controlled trials (RCTs) (22-24); however, there are relatively recent findings indicating that medication reviews conducted by clinical pharmacists on the ward can reduce drug-related readmissions to hospital in various groups of elderly people (25-27). Post-discharge interventions involving phone-based follow-ups by clinical pharmacists have reduced all-cause readmission rates, but these trials did not specifically target an elderly population (28, 29). In addition, there seems to be a need for more RCTs that target medication discrepancies and errors during care transitions between hospital and primary care (30). In summary, there is still insufficient evidence for an optimal post-discharge intervention model that incorporates adherence elements and specifically addresses transitions of care involving elderly people.

In the present study, our primary aim is to investigate the effect of clinical pharmacists in transitional care on unplanned drug-related readmissions among people ≥75 years of age in a context where medication reviews conducted by clinical pharmacists within the hospital are already common practice. Our primary objective is to assess if intervention by a clinical pharmacist during a transition of care can reduce the risk of unplanned drug-related hospital readmission for 180 days after the person leaves the hospital. As secondary objectives, we will study the effects of the intervention on unplanned drug-related readmissions within 30 days, unplanned hospital visits (all-cause readmissions and emergency department visits), mortality, medication adherence, and quality of life. Subgroup analyses will be based on the occurrence of heart failure and cognitive impairment. Moreover, we will collect qualitative interview data regarding the intervention.

Methods and analysis

Principal study design

This study is conducted as a randomised, controlled, superiority trial with two parallel groups. The control group will be assigned to routine care while the intervention group will receive an extended clinical pharmacist service for a period of 180 days, a procedure that incorporates regular medical

For peer review only

chart reviews, medication interviews, medication reviews (**Table 1**), and collaboration with the primary care physician to address DRPs. It should be noted that study participants in both groups may already have been the subject of a medication review performed by a clinical pharmacist during their hospital stay.

Table 1. Important aspects of the medication reviews conducted by clinical pharmacists employed by Region Västerbotten.

**Medication reconciliation**	The pharmacist ensures that the medication records are updated and accurate. This assessment is based on both hospital and primary care medical records
**Overall review of list of medications**	This includes indications for therapies, correct choice of drugs, dosages, treatment duration, and untreated indications
**Clinical symptoms in relation to drug treatment**	Symptoms of ADRs*
**Impaired body function**	Liver and renal function, swallowing difficulties, contraindications and allergies
**Drugs that require specific attention**	Toxic drugs, drugs commonly associated with side-effects, PIMs**
**Interactions**	Drug-drug interactions, drug-food interactions

*ADR = Adverse Drug Reaction **PIM = Potentially Inappropriate Medication

Study population, recruitment, and randomisation

The recruitment of study participants started in September 2019 and takes place among emergency admissions to the University Hospital of Umeå. We will continuously recruit participants until we reach the target sample size of 700 randomised individuals. Thus far, recruitment has been limited to one medical ward; however, it is possible to increase the number of wards if the inclusion rate proves insufficient to reach the target sample size during 2022. People who meet the eligibility criteria (**Table 2**) are given the opportunity to participate in the trial. Once written consent has been obtained (approval from next of kin in cases of people with major NCD), the participants report self-assessed adherence and quality of life through questionnaires. Following discharge from the ward, the participants are randomly assigned to one of the two study arms through a stratified randomisation procedure (**Figure 1**) based on their results from a shortened 4-item version (31) of the Gottfries'

For peer review only

Cognitive Scale, a validated tool for proxy rating cognitive impairment (32, 33). Within each stratum, the participants are further assigned to either of the study arms with an intended ratio of 1:1 through random allocation.

Table 2. List of eligibility criteria

**Inclusion criteria**
• ≥75 years<br>• Living at home (i.e. not in nursing home)<br>• Emergency admission<br>• Registered at one of nine specified primary care centres
**Exclusion criteria**
• Do not speak Swedish or unable to communicate<br>• Admitted due to intoxication (drug or alcohol)<br>• Scheduled for palliative care

Allocation concealment and blinding

We enrol participants before they are discharged from hospital. The allocation sequences are computer generated and managed by external administrators who irreversibly assign the participants to their respective treatments. From this stage onwards, the clinical pharmacists are aware of the group assignments. Study participants are considered blinded until the first phone call. The statistical analysis will be performed in agreement with a statistician who is unaware of the assigned treatments. All information about group assignments will also be unknown to the outcome assessors and any notes in medical records that might offer such clues will be removed beforehand.

Intervention group protocol

Our intervention model (**Figure 2**) incorporates both phone-based interaction with the participants and access to both hospital and primary care medical records. A Master of Pharmacy or similar qualification is required for all clinical pharmacists performing the intervention. The clinical

For peer review only

pharmacist initially contacts the participants within seven days of their discharge from hospital. Other phone-based interviews are scheduled at 30 and 60 days after hospital discharge. Due to practical considerations, interviews may be rearranged to the nearest possible date. In brief, these conversations focus on misunderstands regarding new and ongoing treatments, adherence to medication, and other potential DRPs, ranging from side effects to practical administration barriers.

In addition to the pre-scheduled interviews, the intervention includes repeated medical chart reviews performed on average every second week. This activity is implemented in order to identify discrepancies between the lists of medications before, during, and after hospitalisation, unanswered referrals between healthcare providers, deviating laboratory values, and whether the participant has had any relevant contacts with the healthcare system (**Figure 2**). In the event of new medications or any documented symptoms that might be drug-related, the clinical pharmacist conducts a medication review (**Table 1**). Identified or suspected DRPs that are detected through the interviews or medical chart reviews but cannot be solved by direct communication with the participant or next of kin are instead discussed with the primary care physician. This is the person who ultimately decides on any changes to medication regimens; in which case, the clinical pharmacist contacts the patient within a few days in order to confirm and reinforce the physician´s instructions.

Public and patient involvement

There are no elements of public and patient involvement in the design and conduct of this trial, and we have no current plans of involving patients or the public in the dissemination of the research findings.

Data collection and outcomes

All outcome data will be gathered from medical charts with the exception of self-report questionnaires. All collected research data will be coded and kept in locked archives. Before, during, and after the trial, all forms and protocols used during the interventions and follow-ups that reveal individual personal data are stored according to the same principle. There will be no identifiable data in the dissemination of the results. In summary, outcomes will be measured at the time of discharge

For peer review only

from hospital (baseline) and then after 30 and 180 days respectively (**Table 3**). Rehabilitation needs require that some enrolled participants are transferred to the geriatric department before leaving the hospital for home. If a participant is relocated to another clinical ward, the actual discharge from hospital will be considered as baseline, with the exception of self-reported adherence and quality of life, which are reported on the original ward. As a consequence, we contact all study participants by phone at 30 and 180 days after their first report regarding MARS-5 and EQ-5D, even if this date differs from the actual date on which they were discharged from hospital.

Table 3. Scheduled data collection for primary and secondary outcomes. Baseline data are collected from the date of hospital discharge. The data for MARS-5 and EQ-5D can be retrieved before the participants are discharged and the date on which questionnaires are completed are considered as baseline regarding these two outcomes.

	Baseline	30 days	180 days
**Clinical outcomes**			
Drug-related readmissions		x	x
All-cause readmissions		x	x
Emergency department visits		x	x
Mortality		x	x
**Adherence**			
MARS-5*	x	x	x
**Quality of life**			
EQ-5D**	x	x	x
**Patient characteristics**			
Age and sex	x		
Cohabitant and home healthcare	x		
Diagnoses	x		
Number of medications	x		

*MARS-5 = Medication Adherence Report Scale **EQ-5D = EuroQuol five-dimension

For peer review only

Hospital readmissions

The primary outcome is time until unplanned drug-related readmission within 180 days of being discharged from the hospital. As a secondary outcome, we will also study these events within a narrower timeframe of 30 days. The cause of each readmission will be retrospectively determined by a group of blinded assessors who are not otherwise involved in the study. The initial idea was to rely on the consensus decisions from an expert group of physicians and pharmacists who will judge the causality of each readmission according to probability criteria from WHO, a method that has been used previously in similar studies (25, 34, 35). There are, however, recent examples of validated classification systems with good inter-rater reliability that might be less time-consuming (36, 37) and we will therefore conduct a final review of available options before the assessment process commences in order to find a valid and feasible classification tool. We will also measure time until unplanned hospital visit (all-cause readmissions and emergency department visits), time until death, as well as frequency of unplanned drug-related hospital readmissions and unplanned hospital visits. Outcomes will mainly be analysed through an intention-to-treat (ITT) approach, although we will also conduct per-protocol analyses among those who have received all parts of the intervention.

Medication adherence

Study participants self-report medication adherence using the Medication Adherence Report Scale (MARS-5), a self-assessment questionnaire with five items on a five-point scale that has previously been translated into Swedish (38, 39).

Quality of life and health economics

Quality of life is reported through a Swedish translation of the EuroQuol five-dimension (EQ-5D) questionnaire, a standardised instrument consisting of two different components (40). In the first part, the respondents describe their health status in five different dimensions: walking ability, self-care, usual activities, pain/discomfort, and anxiety/depressive symptoms. This part is followed by a visual analogue scale (EQ-VAS) on which the respondents evaluate their own health status on a scale of 0 to 100.

For peer review only - http://bmjopen.bmj.com/site/about/guidelines.xhtml

Baseline characteristics

Baseline data include various personal characteristics, such as age, sex, living conditions in terms of cohabitation and home health care, current list of medications, diagnoses, and laboratory values.

Sustained use and system-wide implementation

In addition to quantitative data, there will also be a qualitative aspect to the study. Healthcare staff and participants (and in some cases their next of kin) involved in the intervention will be interviewed about the functionalities of the model. An interview guide will be used and the questions will mainly focus on what does and does not work, overall satisfaction, and personal experiences. In addition, interviews will look at potential improvements, refinements to support 'non-adopters' and future implementation, as well as the opportunity to offer other comments and suggestions.

Discontinuations and deviations from the protocol

An enrolled study participant can at any time withdraw consent to participate in the study or simply request that remaining phone calls be cancelled. Furthermore, participants can be excluded for a number of reasons; for example, a change of living circumstances from home to residential care, or unanswered phone-calls. Outcomes will be analysed according to an intention-to-treat principle where all randomised individuals provide outcome data for the complete follow up-period. Any recorded death or withdrawn consent will be censored in the survival analysis from that point and onwards. For the other outcomes, we will apply the last observation carried forward in cases of missing data. Finally, there will be a supplementary per-protocol analysis of drug-related readmissions and questionnaires among participants who have received all pre-scheduled interviews during the follow-up period.

Statistics

For a relative risk reduction of 40% in drug-related hospital readmissions from 19% to 11%, a total study population of 700 individuals is required to achieve statistical power of 80% at our pre-specified significance level of 0.05 for two-sided tests. The estimated incidence of drug-related readmissions

For peer review only

was observed in a previous trial within the same community, although that study was limited to elderly people with major NCD (25).

We will present time-to-event data in Kaplan-Meier survival curves and analyse differences in survival data between the groups through log rank tests and Cox proportional-hazard models. Further investigations of subgroup effects and adjustments of the regression models will be based on heart failure and cognitive impairment as prognostic factors. We will analyse secondary outcomes using various appropriate statistical tests, such as chi-square and independent sample t-test, depending on the type of variable.

Current trial status

The recruitment and enrolment of participants started in September 2018 and there were 20 people enrolled in the trial by the end of that year. From January 2019 onwards, additional staff were involved in the recruitment process and the inclusion rate has since increased. The collection of data is currently ongoing, and 161 people had been randomised by November 2019. The intervention, or phone-based follow-ups in the case of controls, had thus far been discontinued for 44 of these individuals. The main reasons were withdrawn consent (n=13), transfers to nursing homes (n=12), repeated unanswered phone calls (n=9), and death (n=5).

Ethics and dissemination

Prior to hospital discharge, people who fulfil the inclusion criteria are given oral and written information about the study on the clinical ward. Anyone who does not wish to participate is free to decline or withdraw at any time during the course of the trial. Individuals with a major NCD participate without formal written informed consent in accordance with Swedish Ethical Review Authority regulations, although in these cases both the intended participant and the next of kin are informed about the study and given the opportunity to decline participation. The study has been approved by the Regional Ethical Review Board in Umeå (registration numbers 2017-69-31M, 2018-83-32M, and 2018-254-32M). No participant in this trial is subject to any clinical procedures that are likely to be harmful and there are therefore no routines for collecting, assessing, reporting, or

For peer review only

managing adverse events or other unintended effects of the intervention. Moreover, we assume that any participants who are uncomfortable with the trial will exercise their right to withdraw from the protocols. There are no agreements or other regulations that will limit our access to collected data. The study will be reported according to the Consolidated Standards of Reporting Trials (CONSORT) guidelines (41). Our intention is to publish the results with open access in international peer-reviewed journals. We also aim to present the results of the trial at both national and international conferences. The trial is expected to result in more than one published article and form part of two PhD-theses. The study has been registered at ClinicalTrials.gov (trial registration number ID NCT03671629). Any future amendments to this protocol will be recorded in this database.

For peer review only

References

1. Alhawassi TM, Krass I, Bajorek BV, *et al*. A systematic review of the prevalence and risk factors for adverse drug reactions in the elderly in the acute care setting. *Clin Interv Aging*. 2014;9:2079-86.
2. Patel H, Bell D, Molokhia M, *et al*. Trends in hospital admissions for adverse drug reactions in England: analysis of national hospital episode statistics 1998-2005. *BMC Clin Pharmacol*. 2007;7:9.
3. Wittenberg R, Sharpin L, McCormick B, *et al*. The ageing society and emergency hospital admissions. *Health Policy*. 2017;121:923-28.
4. Beijer HJ, de Blaey CJ. Hospitalisations caused by adverse drug reactions (ADR): a meta-analysis of observational studies. *Pharm World Sci*. 2002;24:46-54.
5. Salvi F, Marchetti A, D'Angelo F, *et al*. Adverse drug events as a cause of hospitalization in older adults. *Drug Saf*. 2012;35:29-45.
6. Chan M, Nicklason F, Vial JH. Adverse drug events as a cause of hospital admission in the elderly. *Intern Med J*. 2001;31:199-205.
7. McLachlan CY, Yi M, Ling A, *et al*. Adverse drug events are a major cause of acute medical admission. *Intern Med J*. 2014;44:633-38.
8. Gustafsson M, Sjolander M, Pfister B, *et al*. Drug-related hospital admissions among old people with dementia. *Eur J Clin Pharmacol*. 2016;72:1143-53.
9. Forster AJ, Murff HJ, Peterson JF, *et al*. The incidence and severity of adverse events affecting patients after discharge from the hospital. *Ann Intern Med*. 2003;138:161-67.
10. Moore C, Wisnivesky J, Williams S, *et al*. Medical errors related to discontinuity of care from an inpatient to an outpatient setting. *J Gen Intern Med*. 2003;18:646-51.
11. Perez T, Moriarty F, Wallace E, *et al*. Prevalence of potentially inappropriate prescribing in older people in primary care and its association with hospital admission: longitudinal study. *Bmj*. 2018;363:k4524.
12. World Health Organization. Transitions of Care: Technical Series on Safer Primary Care. Geneva: World Health Organization 2016.
13. Haynes RB, Montague P, Oliver T, *et al*. Interventions for helping patients to follow prescriptions for medications. *Cochrane Database Syst Rev*. 2000:Cd000011.

14. Stewart RB, Caranasos GJ. Medication compliance in the elderly. *Med Clin North Am*. 1989;73:1551-63.

15. Hayes TL, Larimer N, Adami A, *et al*. Medication adherence in healthy elders: small cognitive changes make a big difference. *J Aging Health*. 2009;21:567-80.

16. Rosen OZ, Fridman R, Rosen BT, *et al*. Medication adherence as a predictor of 30-day hospital readmissions. *Patient Prefer Adherence*. 2017;11:801-10.

17. Hughes LD, Witham MD. Causes and correlates of 30 day and 180 day readmission following discharge from a Medicine for the Elderly Rehabilitation unit. *BMC Geriatr*. 2018;18:197.

18. Ruppar TM, Cooper PS, Mehr DR, *et al*. Medication Adherence Interventions Improve Heart Failure Mortality and Readmission Rates: Systematic Review and Meta-Analysis of Controlled Trials. *J Am Heart Assoc*. 2016;5.

19. The definition of clinical pharmacy. *Pharmacotherapy*. 2008;28:816-17.

20. Graabaek T, Kjeldsen LJ. Medication reviews by clinical pharmacists at hospitals lead to improved patient outcomes: a systematic review. *Basic Clin Pharmacol Toxicol*. 2013;112:359-73.

21. Altowaijri A, Phillips CJ, Fitzsimmons D. A systematic review of the clinical and economic effectiveness of clinical pharmacist intervention in secondary prevention of cardiovascular disease. *J Manag Care Pharm*. 2013;19:408-16.

22. Thomas R, Huntley AL, Mann M, *et al*. Pharmacist-led interventions to reduce unplanned admissions for older people: a systematic review and meta-analysis of randomised controlled trials. *Age Ageing*. 2014;43:174-87.

23. Jokanovic N, Tan EC, Sudhakaran S, *et al*. Pharmacist-led medication review in community settings: An overview of systematic reviews. *Res Social Adm Pharm*. 2017;13:661-85.

24. Rankin A, Cadogan CA, Patterson SM, *et al*. Interventions to improve the appropriate use of polypharmacy for older people. *Cochrane Database Syst Rev*. 2018:Cd008165.

25. Gustafsson M, Sjolander M, Pfister B, *et al*. Pharmacist participation in hospital ward teams and hospital readmission rates among people with dementia: a randomized controlled trial. *Eur J Clin Pharmacol*. 2017;73:827-35.

26. Gillespie U, Alassaad A, Henrohn D, *et al*. A comprehensive pharmacist intervention to reduce morbidity in patients 80 years or older: a randomized controlled trial. *Arch Intern Med*. 2009;169:894-900.

27. Lenssen R, Schmitz K, Griesel C, *et al*. Comprehensive pharmaceutical care to prevent drug-related readmissions of dependent-living elderly patients: a randomized controlled trial. *BMC Geriatr*. 2018;18:135.

28. Ravn-Nielsen LV, Duckert ML, Lund ML, *et al*. Effect of an In-Hospital Multifaceted Clinical Pharmacist Intervention on the Risk of Readmission: A Randomized Clinical Trial. *JAMA Intern Med*. 2018;178:375-82.

29. Odeh M, Scullin C, Fleming G, *et al*. Ensuring continuity of patient care across the healthcare interface: Telephone follow-up post-hospitalization. *Br J Clin Pharmacol*. 2019;85:616-25.

30. Kee KW, Char CWT, Yip AYF. A review on interventions to reduce medication discrepancies or errors in primary or ambulatory care setting during care transition from hospital to primary care. *J Family Med Prim Care*. 2018;7:501-06.

31. Lovheim H, Gustafsson M, Isaksson U, *et al*. Gottfries' Cognitive Scale for Staff Proxy Rating of Cognitive Function Among Nursing Home Residents. *J Alzheimers Dis*. 2019;72:1251-60

32. Adolfsson R, Gottfries CG, Nystrom L, *et al*. Prevalence of dementia disorders in institutionalized Swedish old people. The work load imposed by caring for these patients. *Acta Psychiatr Scand*. 1981;63:225-44.

33. Gustafsson M, Isaksson U, Karlsson S, *et al*. Behavioral and psychological symptoms and psychotropic drugs among people with cognitive impairment in nursing homes in 2007 and 2013. *Eur J Clin Pharmacol*. 2016;72:987-94.

For peer review only

34. Mjorndal T, Boman MD, Hagg S, *et al*. Adverse drug reactions as a cause for admissions to a department of internal medicine. *Pharmacoepidemiol Drug Saf*. 2002;11:65-72.
35. Hellstrom LM, Bondesson A, Hoglund P, *et al*. Impact of the Lund Integrated Medicines Management (LIMM) model on medication appropriateness and drug-related hospital revisits. *Eur J Clin Pharmacol*. 2011;67:741-52.
36. Kempen TGH, Hedstrom M, Olsson H, *et al*. Assessment tool for hospital admissions related to medications: development and validation in older patients. *Int J Clin Pharm*. 2019;41:198-206.
37. Thevelin S, Spinewine A, Beuscart JB, *et al*. Development of a standardized chart review method to identify drug-related hospital admissions in older people. *Br J Clin Pharmacol*. 2018;84:2600-14.
38. Mahler C, Hermann K, Horne R, *et al*. Assessing reported adherence to pharmacological treatment recommendations. Translation and evaluation of the Medication Adherence Report Scale (MARS) in Germany. *J Eval Clin Pract*. 2010;16:574-79.
39. Bäck A, Andersson Sundell K, Horne R, *et al*. The Medication Adherence Report Scale (MARS-5) in a Swedish sample with bipolar disorder - a pilot study. *Int J Pers Centered Med*. 2012;2:263-70.
40. Brooks RG, Jendteg S, Lindgren B, *et al*. EuroQol: health-related quality of life measurement. Results of the Swedish questionnaire exercise. *Health Policy*. 1991;18:37-48.
41. Schulz KF, Altman DG, Moher D. CONSORT 2010 Statement: updated guidelines for reporting parallel group randomised trials. *Trials*. 2010;11:32.
42. Editors ICoMJ. Recommendations for the Conduct, Reporting, Editing, and Publication of Scholarly Work in Medical Journals: International Committee of Medical Journal Editors; 2018 [Accessed in November 2019]. Available from: http://www.icmje.org/icmje-recommendations.pdf.

Footnotes

Conflicts of interest

None.

Funding

This study is supported financially by grants from Region Västerbotten, the Swedish Society of Medicine, Umeå University Foundation for Medical Research, Forte (ref: 2017-01438), and the Swedish Research Council (ref: 2019-01078). The funders have no role in the study design, data collection and analysis, decision to publish, or preparation of the manuscript.

For peer review only

Authors´ contributions

JK wrote this study protocol and is involved in the collection of data and as one of the clinical pharmacists performing the intervention. JK and SS are jointly responsible for the enrolment, randomisation, and administration of study participants. MG is the principal investigator and responsible for the funding and overall management of the trial. According to the guidelines issued by the International Committee of Medical Journal Editors (42), all listed authors have been involved in developing or revising the study design, contributed in the review process of this article, approved the final version, and will ensure that issues related to the accuracy or integrity of the work are investigated and resolved.

For peer review only

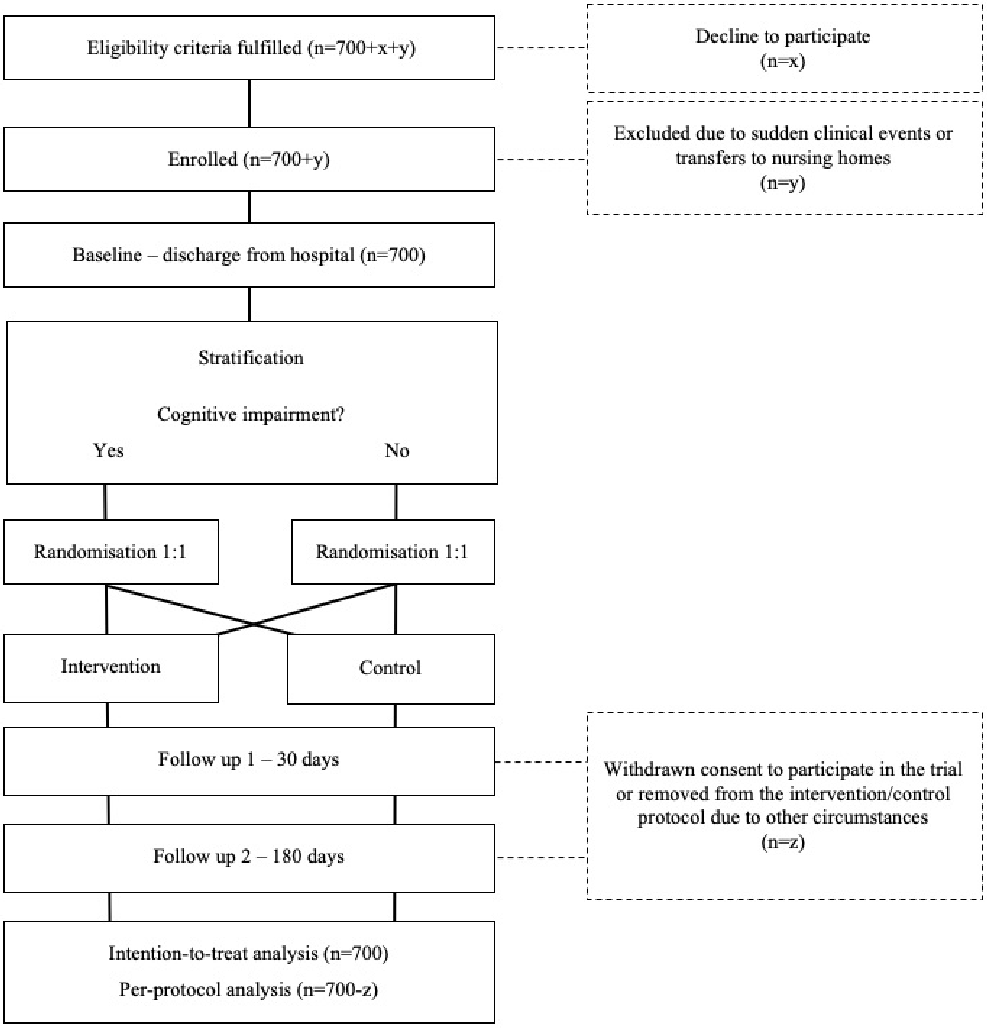

Figure 1. Study flowchart from recruitment to final follow-up.

243x251mm (72 x 72 DPI)

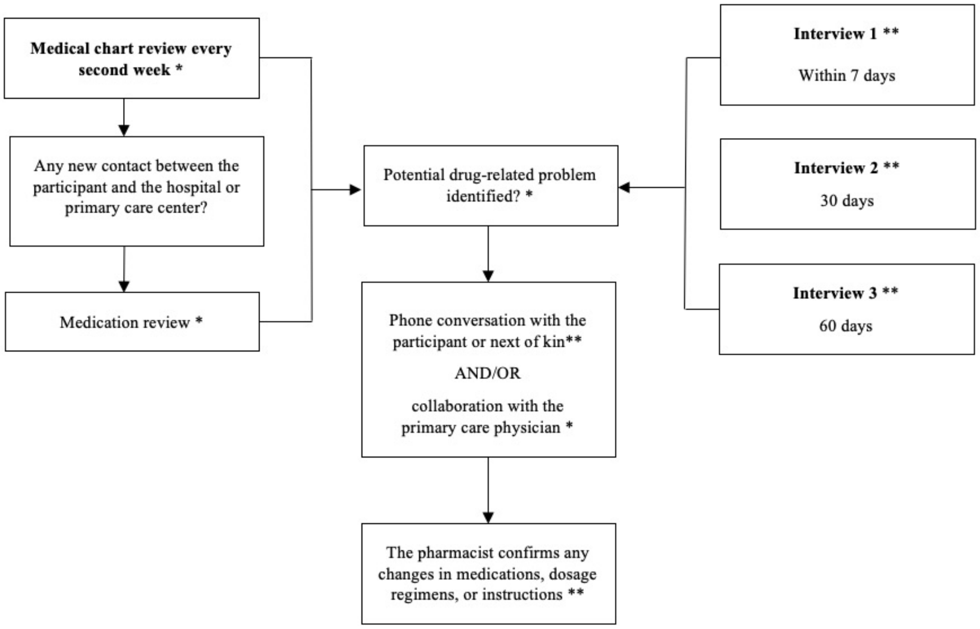

Figure 2. The key features of the intervention, including both pre-scheduled phone interviews and repeated medical chart reviews. The intervention is initiated immediately after hospital discharge. *Clinical pharmacist activity **Interactions between the clinical pharmacist and study participants.

312x201mm (72 x 72 DPI)

SPIRIT

STANDARD PROTOCOL ITEMS: RECOMMENDATIONS FOR INTERVENTIONAL TRIALS

SPIRIT 2013 Checklist: Recommended items to address in a clinical trial protocol and related documents*

Section/item	Item No	Description	Included or mentioned in the protocol
**Administrative information**			
Title	1	Descriptive title identifying the study design, population, interventions, and, if applicable, trial acronym	Yes
Trial registration	2a	Trial identifier and registry name. If not yet registered, name of intended registry	Yes
	2b	All items from the World Health Organization Trial Registration Data Set	No
Protocol version	3	Date and version identifier	Only reference to the registration at ClinicalTrials.gov.
Funding	4	Sources and types of financial, material, and other support	Yes
Roles and responsibilities	5a	Names, affiliations, and roles of protocol contributors	Yes
	5b	Name and contact information for the trial sponsor	Not applicable
	5c	Role of study sponsor and funders, if any, in study design; collection, management, analysis, and interpretation of data; writing of the report; and the decision to submit the report for publication, including whether they will have ultimate authority over any of these activities	Not applicable
	5d	Composition, roles, and responsibilities of the coordinating centre, steering committee, endpoint adjudication committee, data management team, and other individuals or groups overseeing the trial, if applicable (see Item 21a for data monitoring committee)	Not applicable
**Introduction**			

For peer review only

For peer review only - http://bmjopen.bmj.com/site/about/guidelines.xhtml

Background and rationale	6a	Description of research question and justification for undertaking the trial, including summary of relevant studies (published and unpublished) examining benefits and harms for each intervention	Yes
	6b	Explanation for choice of comparators	Yes
Objectives	7	Specific objectives or hypotheses	Yes
Trial design	8	Description of trial design including type of trial (eg, parallel group, crossover, factorial, single group), allocation ratio, and framework (eg, superiority, equivalence, noninferiority, exploratory)	Yes
**Methods: Participants, interventions, and outcomes**			
Study setting	9	Description of study settings (eg, community clinic, academic hospital) and list of countries where data will be collected. Reference to where list of study sites can be obtained	Yes
Eligibility criteria	10	Inclusion and exclusion criteria for participants. If applicable, eligibility criteria for study centres and individuals who will perform the interventions (eg, surgeons, psychotherapists)	Yes
Interventions	11a	Interventions for each group with sufficient detail to allow replication, including how and when they will be administered	Yes
	11b	Criteria for discontinuing or modifying allocated interventions for a given trial participant (eg, drug dose change in response to harms, participant request, or improving/worsening disease)	Yes
	11c	Strategies to improve adherence to intervention protocols, and any procedures for monitoring adherence (eg, drug tablet return, laboratory tests)	Not applicable
	11d	Relevant concomitant care and interventions that are permitted or prohibited during the trial	Yes, see exclusion criteria

For peer review only

Outcomes	12	Primary, secondary, and other outcomes, including the specific measurement variable (eg, systolic blood pressure), analysis metric (eg, change from baseline, final value, time to event), method of aggregation (eg, median, proportion), and time point for each outcome. Explanation of the clinical relevance of chosen efficacy and harm outcomes is strongly recommended	Yes
Participant timeline	13	Time schedule of enrolment, interventions (including any run-ins and washouts), assessments, and visits for participants. A schematic diagram is highly recommended (see Figure)	Yes
Sample size	14	Estimated number of participants needed to achieve study objectives and how it was determined, including clinical and statistical assumptions supporting any sample size calculations	Yes
Recruitment	15	Strategies for achieving adequate participant enrolment to reach target sample size	Yes
**Methods: Assignment of interventions (for controlled trials)**			
Allocation:			
Sequence generation	16a	Method of generating the allocation sequence (eg, computer-generated random numbers), and list of any factors for stratification. To reduce predictability of a random sequence, details of any planned restriction (eg, blocking) should be provided in a separate document that is unavailable to those who enrol participants or assign interventions	Yes
Allocation concealment mechanism	16b	Mechanism of implementing the allocation sequence (eg, central telephone; sequentially numbered, opaque, sealed envelopes), describing any steps to conceal the sequence until interventions are assigned	Yes
Implementation	16c	Who will generate the allocation sequence, who will enrol participants, and who will assign participants to interventions	Yes
Blinding (masking)	17a	Who will be blinded after assignment to interventions (eg, trial participants, care providers, outcome assessors, data analysts), and how	Yes

For peer review only

	17b	If blinded, circumstances under which unblinding is permissible, and procedure for revealing a participant's allocated intervention during the trial	Not applicable
**Methods: Data collection, management, and analysis**			
Data collection methods	18a	Plans for assessment and collection of outcome, baseline, and other trial data, including any related processes to promote data quality (eg, duplicate measurements, training of assessors) and a description of study instruments (eg, questionnaires, laboratory tests) along with their reliability and validity, if known. Reference to where data collection forms can be found, if not in the protocol	Yes
	18b	Plans to promote participant retention and complete follow-up, including list of any outcome data to be collected for participants who discontinue or deviate from intervention protocols	Yes
Data management	19	Plans for data entry, coding, security, and storage, including any related processes to promote data quality (eg, double data entry; range checks for data values). Reference to where details of data management procedures can be found, if not in the protocol	Yes
Statistical methods	20a	Statistical methods for analysing primary and secondary outcomes. Reference to where other details of the statistical analysis plan can be found, if not in the protocol	Yes
	20b	Methods for any additional analyses (eg, subgroup and adjusted analyses)	Yes
	20c	Definition of analysis population relating to protocol non-adherence (eg, as randomised analysis), and any statistical methods to handle missing data (eg, multiple imputation)	Yes
**Methods: Monitoring**			
Data monitoring	21a	Composition of data monitoring committee (DMC); summary of its role and reporting structure; statement of whether it is independent from the sponsor and competing interests; and reference to where further details about its charter can be found, if not in the protocol. Alternatively, an explanation of why a DMC is not needed	No DMC

For peer review only

	21b	Description of any interim analyses and stopping guidelines, including who will have access to these interim results and make the final decision to terminate the trial	Not applicable
Harms	22	Plans for collecting, assessing, reporting, and managing solicited and spontaneously reported adverse events and other unintended effects of trial interventions or trial conduct	Yes
Auditing	23	Frequency and procedures for auditing trial conduct, if any, and whether the process will be independent from investigators and the sponsor	No
**Ethics and dissemination**			
Research ethics approval	24	Plans for seeking research ethics committee/institutional review board (REC/IRB) approval	Yes
Protocol amendments	25	Plans for communicating important protocol modifications (eg, changes to eligibility criteria, outcomes, analyses) to relevant parties (eg, investigators, REC/IRBs, trial participants, trial registries, journals, regulators)	Yes
Consent or assent	26a	Who will obtain informed consent or assent from potential trial participants or authorised surrogates, and how (see Item 32)	Yes
	26b	Additional consent provisions for collection and use of participant data and biological specimens in ancillary studies, if applicable	Not applicable
Confidentiality	27	How personal information about potential and enrolled participants will be collected, shared, and maintained in order to protect confidentiality before, during, and after the trial	Yes
Declaration of interests	28	Financial and other competing interests for principal investigators for the overall trial and each study site	Yes
Access to data	29	Statement of who will have access to the final trial dataset, and disclosure of contractual agreements that limit such access for investigators	Yes
Ancillary and post-trial care	30	Provisions, if any, for ancillary and post-trial care, and for compensation to those who suffer harm from trial participation	No (no provisions)

For peer review only

Dissemination policy	31a	Plans for investigators and sponsor to communicate trial results to participants, healthcare professionals, the public, and other relevant groups (eg, via publication, reporting in results databases, or other data sharing arrangements), including any publication restrictions	Yes
	31b	Authorship eligibility guidelines and any intended use of professional writers	Yes
	31c	Plans, if any, for granting public access to the full protocol, participant-level dataset, and statistical code	No
**Appendices**			
Informed consent materials	32	Model consent form and other related documentation given to participants and authorised surrogates	No, not translated into English
Biological specimens	33	Plans for collection, laboratory evaluation, and storage of biological specimens for genetic or molecular analysis in the current trial and for future use in ancillary studies, if applicable	Not applicable

*It is strongly recommended that this checklist be read in conjunction with the SPIRIT 2013 Explanation & Elaboration for important clarification on the items. Amendments to the protocol should be tracked and dated. The SPIRIT checklist is copyrighted by the SPIRIT Group under the Creative Commons "Attribution-NonCommercial-NoDerivs 3.0 Unported" license.

For peer review only

STUDY PROTOCOL FOR A RANDOMISED CONTROLLED TRIAL INVESTIGATING THE EFFECT OF CLINICAL PHARMACIST INTERVENTION IN TRANSITIONS OF CARE ON DRUG-RELATED HOSPITAL READMISSIONS AMONG THE ELDERLY

Journal:	*BMJ Open*
Manuscript ID	bmjopen-2019-036650.R1
Article Type:	Protocol
Date Submitted by the Author:	21-Feb-2020

Complete List of Authors:	Kindstedt, Jonas; Umeå University, Department of Integrative Medical Biology<br>Svahn, Sofia; Umeå University , Department of Integrative Medical Biology<br>Sjölander, Maria; Umeå University , Department of Integrative Medical Biology<br>Glader, E-L; Umeå University, Department of Public Health and Clinical Medicine<br>Lövheim, Hugo; Umeå University , Department of Community Medicine and Rehabilitation<br>Gustafsson, Maria; Umeå University, Department of Integrative Medical Biology

**Primary Subject Heading**:	Geriatric medicine
Secondary Subject Heading:	Pharmacology and therapeutics
Keywords:	GERIATRIC MEDICINE, CLINICAL PHARMACOLOGY, Clinical trials < THERAPEUTICS

SCHOLARONE™
Manuscripts

I, the Submitting Author has the right to grant and does grant on behalf of all authors of the Work (as defined in the below author licence), an exclusive licence and/or a non-exclusive licence for contributions from authors who are: i) UK Crown employees; ii) where BMJ has agreed a CC-BY licence shall apply, and/or iii) in accordance with the terms applicable for US Federal Government officers or employees acting as part of their official duties; on a worldwide, perpetual, irrevocable, royalty-free basis to BMJ Publishing Group Ltd ("BMJ") its licensees and where the relevant Journal is co-owned by BMJ to the co-owners of the Journal, to publish the Work in this journal and any other BMJ products and to exploit all rights, as set out in our licence.

The Submitting Author accepts and understands that any supply made under these terms is made by BMJ to the Submitting Author unless you are acting as an employee on behalf of your employer or a postgraduate student of an affiliated institution which is paying any applicable article publishing charge ("APC") for Open Access articles. Where the Submitting Author wishes to make the Work available on an Open Access basis (and intends to pay the relevant APC), the terms of reuse of such Open Access shall be governed by a Creative Commons licence – details of these licences and which Creative Commons licence will apply to this Work are set out in our licence referred to above.

Other than as permitted in any relevant BMJ Author's Self Archiving Policies, I confirm this Work has not been accepted for publication elsewhere, is not being considered for publication elsewhere and does not duplicate material already published. I confirm all authors consent to publication of this Work and authorise the granting of this licence.

For peer review only

STUDY PROTOCOL FOR A RANDOMISED CONTROLLED TRIAL INVESTIGATING THE EFFECT OF CLINICAL PHARMACIST INTERVENTION IN TRANSITIONS OF CARE ON DRUG-RELATED HOSPITAL READMISSIONS AMONG THE ELDERLY

Jonas Kindstedt,[1] Sofia Svahn,[1] Maria Sjölander,[1] Eva-Lotta Glader,[2] Hugo Lövheim,[3] Maria Gustafsson[1]

Corresponding author

Jonas Kindstedt, Department of Integrative Medical Biology, Umeå University, Umeå, Sweden, SE-901 87 Umeå, Sweden. Phone: +46 70 657 24 22; E-mail: jonas.kindstedt@umu.se

Word count:

3,082 words

Abstract

Introduction

Drug-related problems (DRPs) are a major cause of unplanned hospital admissions among elderly people, and transitions of care has been emphasised as a key area for improving patient safety. We have designed a complex clinical pharmacist intervention that targets people ≥75 years of age undergoing transitions of care from hospital to home and primary care. The main objective is to investigate if the intervention can reduce the risk of unplanned drug-related readmission within the first 180 days after the person is discharged from hospital.

[1] Department of Integrative Medical Biology, Umeå University, Umeå, Sweden
[2] Department of Public Health and Clinical Medicine, Umeå University, Umeå, Sweden
[3] Department of Community Medicine and Rehabilitation, Umeå University, Umeå, Sweden

For peer review only - http://bmjopen.bmj.com/site/about/guidelines.xhtml
For peer review only

Methods and analysis

This is a randomised, controlled, superiority trial with two parallel arms. A total of 700 people ≥75 years will be assigned to either intervention or routine care (control). The intervention, which aims to find and manage DRPs, is initiated within a week of the person being discharged from hospital and combines repeated medical chart reviews, phone interviews and in some cases medication reviews. People in both study arms may have been the subject of a medication review during their ward stay. As the primary outcome, we will measure time until unplanned drug-related readmission within 180 days of leaving hospital and use log rank tests and Cox proportional-hazard models to analyse differences between the groups. Further investigations of subgroup effects and adjustments of the regression models will be based on heart failure and cognitive impairment as prognostic factors.

Ethics and dissemination

The study has been approved by the Regional Ethical Review Board in Umeå (registration numbers 2017-69-31M, 2018-83-32M and 2018-254-32M) and registered at ClinicalTrials.gov (trial registration number ID NCT03671629). We intend to publish the results with open access in international peer-reviewed journals and present our findings at international conferences. The trial is expected to result in more than one published article and form part of two PhD-theses.

Trial registration number

NCT03671629

Strengths and limitations of this study

- One of few multifaceted clinical pharmacist interventions directed at transitions of care.
- Targets elderly people ≥75 years of age, a vulnerable group rarely addressed in clinical research.
- Conducted in a setting where medication reviews by clinical pharmacists are already implemented during routine hospital care.
- The participants are not blinded to the treatment assignments

For peer review only

Introduction

An ageing population with multiple morbidities and medications brings with it considerable challenges for healthcare and society in terms of adverse drug reactions and other drug-related problems (DRPs).(1) Hospital admission rates increase with age and,(2-4) although prevalence estimates differ between studies,(5) it has been observed that up to 30% of unplanned hospitalisations among the elderly are caused by DRPs,(6, 7) a percentage that appears to be even higher in the vulnerable subgroup of old people with major neurocognitive disorder (NCD).(8) The transition of care between different healthcare providers has been associated with a high risk of inappropriate prescribing, medication errors and adverse drug events,(9-11) and the World Health Organisation (WHO) has identified transitions of care as a key issue to improve patient safety in primary healthcare.(12)

According to review data, poor adherence is a major concern in developed countries given that approximately 50% of all people with medications (all age groups combined) do not follow their recommended therapy.(13) The mechanisms of adherence are multifactorial and, even though there is little evidence to support old age alone as an independent predictor of non-adherent behaviour, the elderly are still at higher risk due to multiple morbid conditions and polypharmacy.(14) Importantly, it has also been observed that even milder forms of cognitive impairment severely affect adherence to medication.(15) In the context of transitions of care, poor adherence in the sensitive post-discharge phase has been associated with hospital readmission and appears to be of particular clinical relevance among people suffering heart failure (16-18).

The clinical pharmacist is one of many professions involved in preventing and managing DRPs. In principle, clinical pharmacy can be described as the science and practice of rational medication use and includes both medication reviews and counselling with patients as well as other health care professionals.(19) Medication reviews conducted by clinical pharmacists have demonstrated a positive overall effect on both the appropriateness and cost of medication.(20, 21) Regarding the effect on hospital readmissions and other clinical outcomes, review data are less consistent and incorporate few randomised controlled trials (RCTs);(22-24) however, there are relatively recent findings indicating that medication reviews conducted by clinical pharmacists on the ward can reduce drug-related readmissions

For peer review only - http://bmjopen.bmj.com/site/about/guidelines.xhtml
For peer review only

to hospital in various groups of elderly people.(25-27) Post-discharge interventions involving phone-based follow-ups by clinical pharmacists have reduced all-cause readmission rates, but these trials did not specifically target an elderly population.(28, 29) In addition, there seems to be a need for more RCTs that target medication discrepancies and errors during care transitions between hospital and primary care.(30) In summary, there is still insufficient evidence for an optimal post-discharge intervention model that incorporates adherence elements and specifically addresses transitions of care involving elderly people.

In the present study, our primary aim is to investigate the effect of clinical pharmacists in transitional care on unplanned drug-related readmissions among people ≥75 years of age in a context where medication reviews conducted by clinical pharmacists within the hospital are already common practice. Our primary objective is to assess if intervention by a clinical pharmacist during a transition of care can reduce the risk of unplanned drug-related hospital readmission for 180 days after the person leaves the hospital. As secondary objectives, we will study the effects of the intervention on unplanned drug-related readmissions within 30 days, unplanned hospital visits (all-cause readmissions and emergency department visits), mortality, medication adherence and quality of life. Subgroup analyses will be based on the occurrence of heart failure and cognitive impairment. Moreover, we will collect qualitative interview data regarding the intervention.

Methods and analysis

Principal study design

This study is conducted as a randomised, controlled, superiority trial with two parallel groups. The control group will be assigned to routine care while the intervention group will receive an extended clinical pharmacist service for a period of 180 days, a procedure that incorporates regular medical chart reviews, medication interviews, medication reviews (**Table 1**) and collaboration with the primary care physician to address DRPs. It should be noted that study participants in both groups may already have been the subject of a medication review performed by a clinical pharmacist during their hospital stay.

For peer review only - http://bmjopen.bmj.com/site/about/guidelines.xhtml

Table 1. Important aspects of the medication reviews conducted by clinical pharmacists employed by Region Västerbotten.

**Medication reconciliation**	The pharmacist ensures that the medication records are updated and accurate. This assessment is based on both hospital and primary care medical records
**Overall review of list of medications**	This includes indications for therapies, correct choice of drugs, dosages, treatment duration and untreated indications
**Clinical symptoms in relation to drug treatment**	Symptoms of ADRs*
**Impaired body function**	Liver and renal function, swallowing difficulties, contraindications and allergies
**Drugs that require specific attention**	Toxic drugs, drugs commonly associated with side-effects and PIMs**
**Interactions**	Drug-drug interactions and drug-food interactions

*ADR = Adverse Drug Reaction **PIM = Potentially Inappropriate Medication

Study population, recruitment and randomisation

The recruitment of study participants started in September 2019 and takes place among emergency admissions to the University Hospital of Umeå. We will continuously recruit participants until we reach the target sample size of 700 randomised individuals. Thus far, recruitment has been limited to one medical ward; however, it is possible to increase the number of wards if the inclusion rate proves insufficient to reach the target sample size during 2022. People who meet the eligibility criteria are given the opportunity to participate in the trial (**Table 2**). Once written consent has been obtained (approval from next of kin in cases of people with major NCD), the participants report self-assessed adherence and quality of life through questionnaires. Following discharge from the ward, the participants are randomly assigned to one of the two study arms through a stratified randomisation procedure (**Figure 1**) based on their results from a shortened 4-item version of the Gottfries' Cognitive Scale,(31) a validated tool for proxy rating cognitive impairment.(32, 33) Within each stratum, the participants are further assigned to either of the study arms with an intended ratio of 1:1 through random allocation.

For peer review only - http://bmjopen.bmj.com/site/about/guidelines.xhtml

Table 2. List of eligibility criteria

**Inclusion criteria**
• ≥75 years<br>• Living at home (i.e. not in nursing home)<br>• Emergency admission<br>• Registered at one of nine specified primary care centres
**Exclusion criteria**
• Do not speak Swedish or unable to communicate<br>• Admitted due to intoxication (drug or alcohol)<br>• Scheduled for palliative care

Allocation concealment and blinding

We enrol participants before they are discharged from hospital. The allocation sequences are computer generated and managed by external administrators who irreversibly assign the participants to their respective treatments. From this stage onwards, the clinical pharmacists are aware of the group assignments. Study participants are considered blinded until the first phone call. The statistical analysis will be performed in agreement with a statistician who is unaware of the assigned treatments. All information about group assignments will also be unknown to the outcome assessors and any notes in medical records that might offer such clues will be removed beforehand.

Intervention group protocol

Our intervention model (**Figure 2**) incorporates both phone-based interaction with the participants and access to both hospital and primary care medical records. A Master of Pharmacy or similar qualification is required for all clinical pharmacists performing the intervention. The clinical pharmacist initially contacts the participants within seven days of their discharge from hospital. Other phone-based interviews are scheduled at 30 and 60 days after hospital discharge. Due to practical considerations, interviews may be rearranged to the nearest possible date. In brief, these conversations focus on

For peer review only - http://bmjopen.bmj.com/site/about/guidelines.xhtml

misunderstands regarding new and ongoing treatments, adherence to medication and other potential DRPs, ranging from side effects to practical administration barriers.

In addition to the pre-scheduled interviews, the intervention includes repeated medical chart reviews performed on average every second week during a period of 180 days after the participants have been discharged from hospital. This activity is implemented in order to identify discrepancies between the lists of medications before, during and after hospitalisation, unanswered referrals between healthcare providers, deviating laboratory values and whether the participant has had any relevant contacts with the healthcare system (**Figure 2**). Moreover, the medical chart reviews also function as an important basis for the interviews. In the event of new medications or any documented signs or symptoms that might be drug-related, the clinical pharmacist conducts a medication review (**Table 1**). This process includes several steps, such as medication reconciliation, evaluation of proper dosage and indication, signs of ADRs or side effects, check for interactions and special considerations due to impaired renal function. In brief, the exact types of DRPs can differ considerably between participants and range from administration issues and poor medication adherence to drug-induced electrolyte imbalance or orthostatic hypotension. Identified or suspected DRPs that are detected through the interviews or medical chart reviews but cannot be solved by direct communication with the participant or next of kin are instead communicated over phone with the primary care physician. In this dialogue, the clinical pharmacist describes the potential DRP being present and offers a recommendation on how to manage the issue. The physician is the person who ultimately decides on any changes to medication regimens; in which case, the clinical pharmacist contacts the patient within a few days in order to confirm and reinforce the physician´s instructions. All clinical pharmacists involved in the trial have electronic access to the participants' medical charts. This access includes physicians' notes, laboratory records, current lists of medications and epicrises. The combined information from these sources provide a basis for both medical chart reviews and medication reviews. During the intervention, the clinical pharmacist continuously documents the time spent on the different activities for each participant.

For peer review only - http://bmjopen.bmj.com/site/about/guidelines.xhtml

Public and patient involvement

There are no elements of public and patient involvement in the design and conduct of this trial, and we have no current plans of involving patients or the public in the dissemination of the research findings.

Data collection and outcomes

All outcome data will be gathered from medical charts with the exception of self-report questionnaires. All collected research data will be coded and kept in locked archives. Before, during and after the trial, all forms and protocols used during the interventions and follow-ups that reveal individual personal data are stored according to the same principle. There will be no identifiable data in the dissemination of the results. In summary, outcomes will be measured at the time of discharge from hospital (baseline) and then after 30 and 180 days respectively (**Table 3**). The follow-ups on MARS-5 and EQ-5D are conducted by phone and preferably in conjunction with the intervention phone calls. Rehabilitation needs require that some enrolled participants are transferred to the geriatric department before leaving the hospital for home. If a participant is relocated to another clinical ward, the actual discharge from hospital will be considered as baseline, with the exception of self-reported adherence and quality of life, which are reported on the original ward. As a consequence, we contact all study participants by phone at 30 and 180 days after their first report regarding MARS-5 and EQ-5D, even if this date differs from the actual date on which they were discharged from hospital.

Table 3. Scheduled data collection for primary and secondary outcomes. Baseline data are collected from the date of hospital discharge. The data for MARS-5 and EQ-5D can be retrieved before the participants are discharged and the date on which questionnaires are completed are considered as baseline regarding these two outcomes.

	**Baseline**	**30 days**	**180 days**
**Clinical outcomes**			
Drug-related readmissions		x	x
All-cause readmissions		x	x
Emergency department visits		x	x

For peer review only - http://bmjopen.bmj.com/site/about/guidelines.xhtml

Mortality		x	x
**Adherence**			
MARS-5*	x	x	x
**Quality of life**			
EQ-5D**	x	x	x
**Patient characteristics**			
Age and sex	x		
Cohabitant and home healthcare	x		
Diagnoses	x		
Number of medications	x		

*MARS-5 = Medication Adherence Report Scale **EQ-5D = EuroQuol five-dimension

For peer review only

Hospital readmissions

The primary outcome is time until unplanned drug-related readmission within 180 days of being discharged from the hospital. As a secondary outcome, we also study these events within a narrower timeframe of 30 days. The cause of each readmission will be retrospectively determined by a group of blinded physicians and pharmacists who are not otherwise involved in the study. This assessment group will reach a consensus decision regarding the causality of each readmission according to criteria from WHO, a method that has been used previously in similar studies.(25, 34, 35) Readmissions classified as *certain*, *probable* and *possible* in this assessment will be regarded as drug-related in the following statistical analysis. We also measure time until unplanned hospital visit (all-cause readmissions and emergency department visits), time until death, as well as frequency of unplanned drug-related hospital readmissions and unplanned hospital visits. Outcomes will mainly be analysed through an intention-to-treat (ITT) approach, although we will also conduct per-protocol analyses among those who have received all parts of the intervention.

Medication adherence

Study participants self-report medication adherence using the Medication Adherence Report Scale (MARS-5), a self-assessment questionnaire with five items on a five-point scale that has previously been translated into Swedish.(36, 37)

Quality of life and health economics

Quality of life is reported through a Swedish translation of the EuroQuol five-dimension (EQ-5D) questionnaire, a standardised instrument consisting of two different components.(38) In the first part, the respondents describe their health status in five different dimensions: walking ability, self-care, usual activities, pain/discomfort and anxiety/depressive symptoms. This part is followed by a visual analogue scale (EQ-VAS) on which the respondents evaluate their own health status on a scale of 0 to 100.

Baseline characteristics

Baseline data include various personal characteristics, such as age, sex, living conditions in terms of cohabitation and home health care, current list of medications, diagnoses and laboratory values.

Sustained use and system-wide implementation

In addition to quantitative data, there will also be a qualitative component of the study. In order to capture the functionalities of the model, healthcare staff and participants involved in the intervention, and in some cases their next of kin, will be interviewed. An interview guide will be used and the questions will mainly focus on what does and does not work, overall satisfaction and personal experiences.(39) The interview guide will also provide the opportunity to ask additional questions and elaborate upon other issues. Moreover, the interviews will explore potential improvements, refinements to support 'non-adopters' and future implementation, as well as the opportunity to offer other comments and suggestions. The interviews will be audio-recorded and transcribed verbatim, and the analysis will be conducted in a systematic process for developing codes and themes in interview data. While coding entails the work of naming the different categories, the thematisation process involves moving from so-called free-floating codes into an integrated framework of empirical themes.(40)

For peer review only - http://bmjopen.bmj.com/site/about/guidelines.xhtml

Discontinuations and deviations from the protocol

An enrolled study participant can at any time withdraw consent to participate in the study or simply request that remaining phone calls be cancelled. Furthermore, the intervention can be discontinued for a number of other reasons, such as a change of living circumstances from home to residential care or repeated unanswered phone-calls. Importantly, we do not continue to record any new information from participants who withdraw their consent to participate in the study. Any documented death or withdrawn consent is censored in the survival analysis from that point and onwards. In all other cases of discontinued interventions, we collect data regarding hospital readmissions and emergency department visits for the complete 180-day period. Outcomes will primarily be analysed according to an intention-to-treat principle that includes all randomised participants for the time they were under observation. For outcomes other than time-to-event, we will apply the last observation carried forward in cases of missing data. Finally, there will be a supplementary per-protocol analysis of drug-related readmissions and questionnaires among participants who have received all pre-scheduled interviews during the follow-up period.

Statistics

For a relative risk reduction of 40% in drug-related hospital readmissions from 19% to 11%, a total study population of 700 individuals is required to achieve statistical power of 80% at our pre-specified significance level of 0.05 for two-sided tests. The estimated incidence of drug-related readmissions was observed in a previous trial within the same community, although that study was limited to elderly people with major NCD.(25)

We will present time-to-event data in Kaplan-Meier survival curves and analyse differences in survival data between the groups through log rank tests and Cox proportional-hazard models. Further investigations of subgroup effects and adjustments of the regression models will be based on heart failure and cognitive impairment as prognostic factors. We will analyse secondary outcomes using various appropriate statistical tests, such as chi-square and independent sample t-test, depending on the type of variable.

For peer review only - http://bmjopen.bmj.com/site/about/guidelines.xhtml

Current trial status

The recruitment and enrolment of participants started in September 2018 and there were 20 people enrolled in the trial by the end of that year. From January 2019 onwards, additional staff were involved in the recruitment process and the inclusion rate has since increased. The collection of data is currently ongoing, and 161 people had been randomised by November 2019. The intervention, or phone-based follow-ups in the case of controls, had thus far been discontinued for 44 of these individuals. The main reasons were withdrawn consent (n=13), transfers to nursing homes (n=12), repeated unanswered phone calls (n=9) and death (n=5).

Limitations and risk of bias

The first and second author of this protocol are involved in the trial both as researchers and as clinical pharmacists conducting the intervention. As previously mentioned, we will undertake several measures to reduce the risk of bias that could emerge from these multiple roles. For example, we will consult external staff for the primary outcome assessment and statistical analyses. Other clinical outcomes in terms of all-cause readmissions, emergency department visits and mortality are electronically documented data with no room for subjectivity. Therefore, this risk of bias mainly applies to MARS-5 and EQ-5D, which should be considered in the interpretation of our findings. Moreover, all interviews within the qualitative part of the study will be conducted by another person than the clinical pharmacist who performed the interventions on those specific participants. Due to the nature of the intervention, neither participants nor clinical pharmacists are blinded. We do not consider the lack of blinding to be a major issue for the study, but bias related to special attention and the knowledge of being observed could possibly affect self-reported adherence and quality of life among the participants.

Ethics and dissemination

Prior to hospital discharge, people who fulfil the inclusion criteria are given oral and written information about the study on the clinical ward. Anyone who does not wish to participate is free to decline or withdraw at any time during the course of the trial. Individuals with a major NCD participate without formal written informed consent in accordance with Swedish Ethical Review Authority regulations,

For peer review only

For peer review only - http://bmjopen.bmj.com/site/about/guidelines.xhtml

although in these cases both the intended participant and the next of kin are informed about the study and given the opportunity to decline participation. The study has been approved by the Regional Ethical Review Board in Umeå (registration numbers 2017-69-31M, 2018-83-32M and 2018-254-32M). No participant in this trial is subject to any clinical procedures that are likely to be harmful and there are therefore no routines for collecting, assessing, reporting or managing adverse events or other unintended effects of the intervention. Moreover, we assume that any participants who are uncomfortable with the trial will exercise their right to withdraw from the protocols. There are no agreements or other regulations that will limit our access to collected data. The study will be reported according to the Consolidated Standards of Reporting Trials (CONSORT) guidelines.(41) Our intention is to publish the results with open access in international peer-reviewed journals. We also aim to present the results of the trial at both national and international conferences. The trial is expected to result in more than one published article and form part of two PhD-theses. In accordance with the guidelines issued by the International Committee of Medical Journal Editors,(42) all listed authors have been involved in developing or revising the study design, contributed in the review process of this article, approved the final version and will ensure that issues related to the accuracy or integrity of the work are investigated and resolved. The study has been registered at ClinicalTrials.gov (trial registration number ID NCT03671629). Any future amendments to this protocol will be recorded in this database.

References

1. Alhawassi TM, Krass I, Bajorek BV, *et al.* A systematic review of the prevalence and risk factors for adverse drug reactions in the elderly in the acute care setting. *Clin Interv Aging*. 92014. p. 2079-86.
2. Patel H, Bell D, Molokhia M, Srishanmuganathan J, *et al.* Trends in hospital admissions for adverse drug reactions in England: analysis of national hospital episode statistics 1998-2005. *BMC Clin Pharmacol*. 2007;7:9.
3. Wittenberg R, Sharpin L, McCormick B, *et al.* The ageing society and emergency hospital admissions. *Health Policy*. 2017;121:923-28.
4. Beijer HJ, de Blaey CJ. Hospitalisations caused by adverse drug reactions (ADR): a meta-analysis of observational studies. *Pharm World Sci*. 2002;24:46-54.
5. Salvi F, Marchetti A, D'Angelo F, *et al.* Adverse drug events as a cause of hospitalization in older adults. *Drug Saf.* 2012;35 Suppl 1:29-45.
6. Chan M, Nicklason F, Vial JH. Adverse drug events as a cause of hospital admission in the elderly. *Intern Med J*. 2001;31:199-205.
7. McLachlan CY, Yi M, Ling A, *et al.* Adverse drug events are a major cause of acute medical admission. *Intern Med J*. 2014;44:633-38.

For peer review only - http://bmjopen.bmj.com/site/about/guidelines.xhtml

8. Gustafsson M, Sjolander M, Pfister B, *et al*. Drug-related hospital admissions among old people with dementia. *Eur J Clin Pharmacol*. 2016;72:1143-53.

9. Forster AJ, Murff HJ, Peterson JF, *et al*. The incidence and severity of adverse events affecting patients after discharge from the hospital. *Ann Intern Med*. 2003;138:161-7.

10. Moore C, Wisnivesky J, Williams S, *et al*. Medical errors related to discontinuity of care from an inpatient to an outpatient setting. *J Gen Intern Med*. 2003;18:646-51.

11. Perez T, Moriarty F, Wallace E, *et al*. Prevalence of potentially inappropriate prescribing in older people in primary care and its association with hospital admission: longitudinal study. *Bmj*. 2018;363:k4524.

12. World Health Organisation. Transitions of Care: Technical Series on Safer Primary Care. Geneva: World Health Organisation; 2016.

13. Haynes RB, Montague P, Oliver T, *et al*. Interventions for helping patients to follow prescriptions for medications. Cochrane Database Syst Rev. 2000(2):Cd000011.

14. Stewart RB, Caranasos GJ. Medication compliance in the elderly. *Med Clin North Am*. 1989;73:1551-63.

15. Hayes TL, Larimer N, Adami A, *et al*. Medication adherence in healthy elders: small cognitive changes make a big difference. *J Aging Health*. 2009;21:567-80.

16. Rosen OZ, Fridman R, Rosen BT, *et al*. Medication adherence as a predictor of 30-day hospital readmissions. *Patient Prefer Adherence*. 2017;11:801-10.

17. Hughes LD, Witham MD. Causes and correlates of 30 day and 180 day readmission following discharge from a Medicine for the Elderly Rehabilitation unit. *BMC Geriatr*. 2018;18:197.

18. Ruppar TM, Cooper PS, Mehr DR, *et al*. Medication Adherence Interventions Improve Heart Failure Mortality and Readmission Rates: Systematic Review and Meta-Analysis of Controlled Trials. *J Am Heart Assoc*. 2016;5.

19. The definition of clinical pharmacy. *Pharmacotherapy*. 2008;28:816-17.

20. Graabaek T, Kjeldsen LJ. Medication reviews by clinical pharmacists at hospitals lead to improved patient outcomes: a systematic review. *Basic Clin Pharmacol Toxicol*. 2013;112:359-73.

21. Altowaijri A, Phillips CJ, Fitzsimmons D. A systematic review of the clinical and economic effectiveness of clinical pharmacist intervention in secondary prevention of cardiovascular disease. *J Manag Care Pharm*. 2013;19:408-16.

22. Thomas R, Huntley AL, Mann M, *et al*. Pharmacist-led interventions to reduce unplanned admissions for older people: a systematic review and meta-analysis of randomised controlled trials. *Age Ageing*. 2014;43(2):174-87.

23. Jokanovic N, Tan EC, Sudhakaran S, *et al*. Pharmacist-led medication review in community settings: An overview of systematic reviews. *Res Social Adm Pharm*. 2017;13(4):661-85.

24. Rankin A, Cadogan CA, Patterson SM, *et al*. Interventions to improve the appropriate use of polypharmacy for older people. *Cochrane Database Syst Rev*. 2018:Cd008165.

25. Gustafsson M, Sjolander M, Pfister B, *et al*. Pharmacist participation in hospital ward teams and hospital readmission rates among people with dementia: a randomized controlled trial. Eur J Clin Pharmacol. 2017;73(7):827-35.

26. Gillespie U, Alassaad A, Henrohn D, *et al*. A comprehensive pharmacist intervention to reduce morbidity in patients 80 years or older: a randomized controlled trial. *Arch Intern Med*. 2009;169:894-900.

27. Lenssen R, Schmitz K, Griesel C, *et al*. Comprehensive pharmaceutical care to prevent drug-related readmissions of dependent-living elderly patients: a randomized controlled trial. *BMC Geriatr*. 2018;18:135.

28. Ravn-Nielsen LV, Duckert ML, Lund ML, *et al*. Effect of an In-Hospital Multifaceted Clinical Pharmacist Intervention on the Risk of Readmission: A Randomized Clinical Trial. *JAMA Intern Med*. 2018;178:375-82.

For peer review only - http://bmjopen.bmj.com/site/about/guidelines.xhtml

29. Odeh M, Scullin C, Fleming G, *et al*. Ensuring continuity of patient care across the healthcare interface: Telephone follow-up post-hospitalization. *Br J Clin Pharmacol*. 2019;85:616-25.

30. Kee KW, Char CWT, Yip AYF. A review on interventions to reduce medication discrepancies or errors in primary or ambulatory care setting during care transition from hospital to primary care. *J Family Med Prim Care*. 2018;7:501-06.

31. Lovheim H, Gustafsson M, Isaksson U, *et al*. Gottfries' Cognitive Scale for Staff Proxy Rating of Cognitive Function Among Nursing Home Residents. *J Alzheimers Dis*. 2019;72:1251-60

32. Adolfsson R, Gottfries CG, Nystrom L, *et al*. Prevalence of dementia disorders in institutionalized Swedish old people. The work load imposed by caring for these patients. *Acta Psychiatr Scand*. 1981;63:225-44.

33. Gustafsson M, Isaksson U, Karlsson S, *et al*. Behavioral and psychological symptoms and psychotropic drugs among people with cognitive impairment in nursing homes in 2007 and 2013. *Eur J Clin Pharmacol*. 2016;72:987-94.

34. Mjorndal T, Boman MD, Hagg S, *et al*. Adverse drug reactions as a cause for admissions to a department of internal medicine. *Pharmacoepidemiol Drug Saf*. 2002;11:65-72.

35. Hellstrom LM, Bondesson A, Hoglund P, *et al*. Impact of the Lund Integrated Medicines Management (LIMM) model on medication appropriateness and drug-related hospital revisits. *Eur J Clin Pharmacol*. 2011;67:741-52.

36. Mahler C, Hermann K, Horne R, *et al*. Assessing reported adherence to pharmacological treatment recommendations. Translation and evaluation of the Medication Adherence Report Scale (MARS) in Germany. *J Eval Clin Pract*. 2010;16:574-79.

37. Bäck A, Andersson Sundell K, Horne R, *et al*. The Medication Adherence Report Scale (MARS-5) in a Swedish sample with bipolar disorder - a pilot study. *Int J Pers Centered Med*. 2012;2:263-70

38. Brooks RG, Jendteg S, Lindgren B, *et al*. EuroQol: health-related quality of life measurement. Results of the Swedish questionnaire exercise. *Health Policy*. 1991;18:37-48

39. Quinn PM. Qualitative research & evaluation methods. London, England: SAGE Publications 2002:342-60.

40. Mikael H, Simon L, Marco N. Introduktion till samhällsvetenskaplig analys [Introduction to Social Scientific Analysis]. Malmö, Sweden: Gleerups Utbildning 2010:114-18

41. Schulz KF, Altman DG, Moher D. CONSORT 2010 Statement: updated guidelines for reporting parallel group randomised trials. *Trials*. 2010;11:32.

42. International Journal of Medical Journal Editors. Recommendations for the Conduct, Reporting, Editing, and Publication of Scholarly Work in Medical Journals: International Committee of Medical Journal Editors; 2018 [Accessed 1 November 2019]. Available from: http://www.icmje.org/icmje-recommendations.pdf.

Footnotes

Conflicts of interest

None.

Funding

This study is supported financially by grants from Region Västerbotten, the Swedish Society of Medicine, Umeå University Foundation for Medical Research, Forte (ref: 2017-01438) and the Swedish

For peer review only - http://bmjopen.bmj.com/site/about/guidelines.xhtml

Research Council (ref: 2019-01078). The funders have no role in the study design, data collection and analysis, decision to publish or preparation of the manuscript.

Authors´ contributions

JK wrote this study protocol. MG, HL, ELG and SS developed the original study design. JK, SS, MS, ELG, HL and MG were all involved in the revision of the study design and contributed in the review process of the protocol manuscript. JK and SS are jointly responsible for the collection of data and administration of study participants. JK and SS are also involved in the trial as two of the clinical pharmacists performing the intervention. MG is the principal investigator and responsible for the funding and overall management of the trial.

List of figure captions

Figure 1. Study flow-chart from recruitment to final follow-up.

Figure 2. The key features of the intervention, including both pre-scheduled phone interviews and repeated medical chart reviews. The intervention is initiated immediately after hospital discharge and goes on for 180 days. *Clinical pharmacist activity **Interactions between clinical pharmacist and study participants

For peer review only

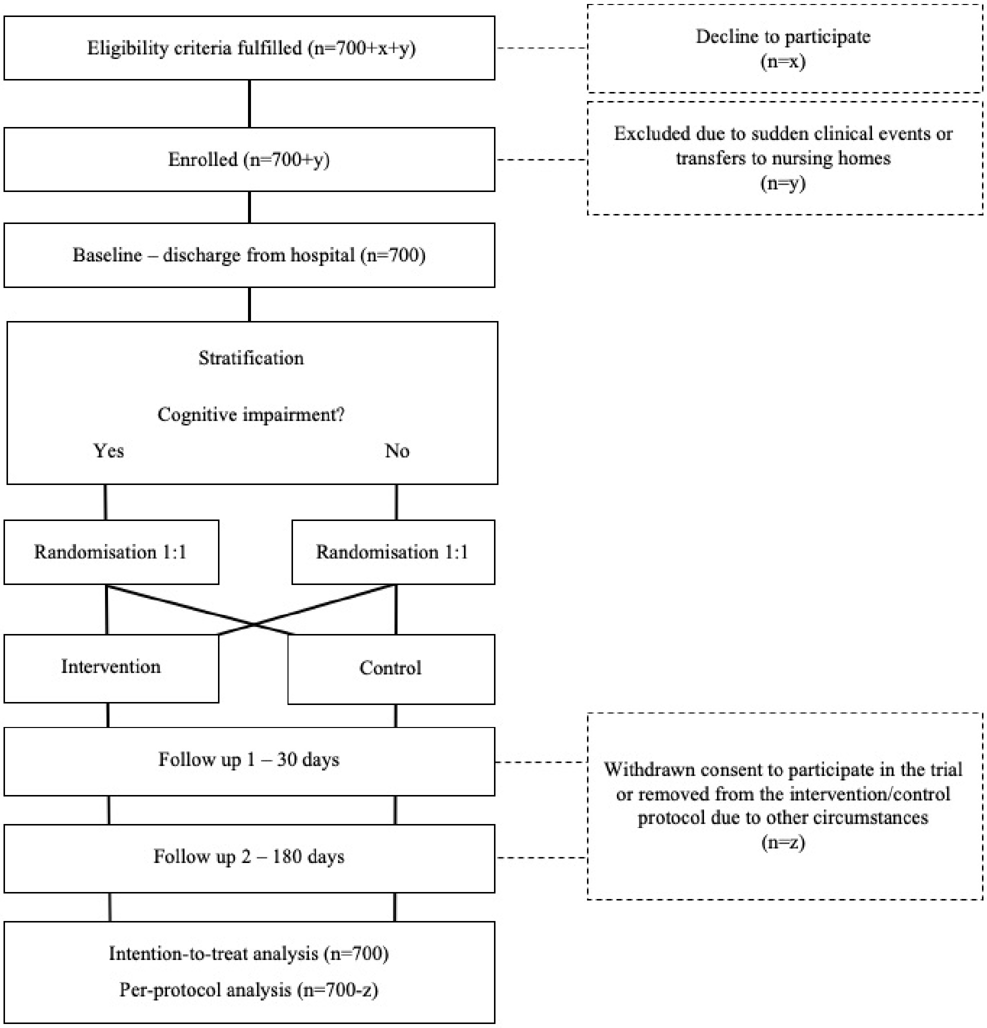

Figure 1. Study flowchart from recruitment to final follow-up.

243x251mm (72 x 72 DPI)

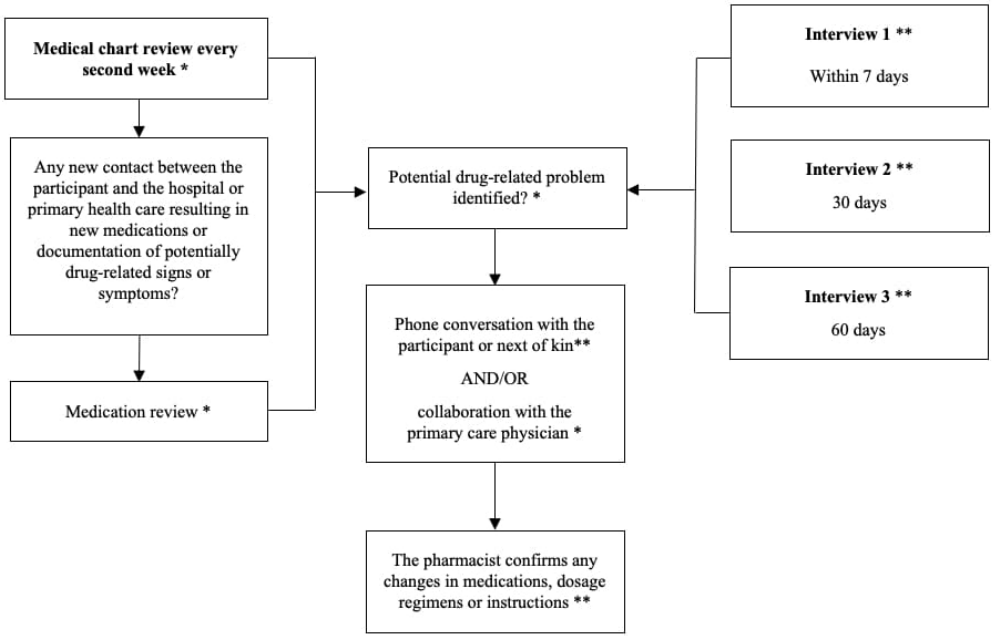

Figure 2. The key features of the intervention, including both pre-scheduled phone interviews and repeated medical chart reviews. The intervention is initiated immediately after hospital discharge and goes on for 180 days. *Clinical pharmacist activity **Interactions between clinical pharmacist and study participants

22910x14782mm (1 x 1 DPI)

SPIRIT

STANDARD PROTOCOL ITEMS: RECOMMENDATIONS FOR INTERVENTIONAL TRIALS

SPIRIT 2013 Checklist: Recommended items to address in a clinical trial protocol and related documents*

Section/item	Item No	Description	Included or mentioned in the protocol
**Administrative information**			
Title	1	Descriptive title identifying the study design, population, interventions, and, if applicable, trial acronym	Page 1
Trial registration	2a	Trial identifier and registry name. If not yet registered, name of intended registry	Page 2 and 13
	2b	All items from the World Health Organization Trial Registration Data Set	n/a
Protocol version	3	Date and version identifier	n/a
Funding	4	Sources and types of financial, material, and other support	Page 16
Roles and responsibilities	5a	Names, affiliations, and roles of protocol contributors	Page 1 and 16
	5b	Name and contact information for the trial sponsor	n/a
	5c	Role of study sponsor and funders, if any, in study design; collection, management, analysis, and interpretation of data; writing of the report; and the decision to submit the report for publication, including whether they will have ultimate authority over any of these activities	n/a
	5d	Composition, roles, and responsibilities of the coordinating centre, steering committee, endpoint adjudication committee, data management team, and other individuals or groups overseeing the trial, if applicable (see Item 21a for data monitoring committee)	n/a
**Introduction**			

For peer review only

Background and rationale	6a	Description of research question and justification for undertaking the trial, including summary of relevant studies (published and unpublished) examining benefits and harms for each intervention	Page 3-4
	6b	Explanation for choice of comparators	Page 4-5
Objectives	7	Specific objectives or hypotheses	Page 4
Trial design	8	Description of trial design including type of trial (eg, parallel group, crossover, factorial, single group), allocation ratio, and framework (eg, superiority, equivalence, noninferiority, exploratory)	Page 4
**Methods: Participants, interventions, and outcomes**			
Study setting	9	Description of study settings (eg, community clinic, academic hospital) and list of countries where data will be collected. Reference to where list of study sites can be obtained	Page 5
Eligibility criteria	10	Inclusion and exclusion criteria for participants. If applicable, eligibility criteria for study centres and individuals who will perform the interventions (eg, surgeons, psychotherapists)	Page 6
Interventions	11a	Interventions for each group with sufficient detail to allow replication, including how and when they will be administered	Page 6-7
	11b	Criteria for discontinuing or modifying allocated interventions for a given trial participant (eg, drug dose change in response to harms, participant request, or improving/worsening disease)	Page 11
	11c	Strategies to improve adherence to intervention protocols, and any procedures for monitoring adherence (eg, drug tablet return, laboratory tests)	n/a
	11d	Relevant concomitant care and interventions that are permitted or prohibited during the trial	Page 6

For peer review only

Outcomes	12	Primary, secondary, and other outcomes, including the specific measurement variable (eg, systolic blood pressure), analysis metric (eg, change from baseline, final value, time to event), method of aggregation (eg, median, proportion), and time point for each outcome. Explanation of the clinical relevance of chosen efficacy and harm outcomes is strongly recommended	Page 8-10
Participant timeline	13	Time schedule of enrolment, interventions (including any run-ins and washouts), assessments, and visits for participants. A schematic diagram is highly recommended (see Figure)	Page 5-7
Sample size	14	Estimated number of participants needed to achieve study objectives and how it was determined, including clinical and statistical assumptions supporting any sample size calculations	Page 5 and 11
Recruitment	15	Strategies for achieving adequate participant enrolment to reach target sample size	Page 5 and 12
**Methods: Assignment of interventions (for controlled trials)**			
Allocation:			
Sequence generation	16a	Method of generating the allocation sequence (eg, computer-generated random numbers), and list of any factors for stratification. To reduce predictability of a random sequence, details of any planned restriction (eg, blocking) should be provided in a separate document that is unavailable to those who enrol participants or assign interventions	Page 5-6
Allocation concealment mechanism	16b	Mechanism of implementing the allocation sequence (eg, central telephone; sequentially numbered, opaque, sealed envelopes), describing any steps to conceal the sequence until interventions are assigned	Page 6
Implementation	16c	Who will generate the allocation sequence, who will enrol participants, and who will assign participants to interventions	Page 6
Blinding (masking)	17a	Who will be blinded after assignment to interventions (eg, trial participants, care providers, outcome assessors, data analysts), and how	Page 6

For peer review only

	17b	If blinded, circumstances under which unblinding is permissible, and procedure for revealing a participant's allocated intervention during the trial	n/a
**Methods: Data collection, management, and analysis**			
Data collection methods	18a	Plans for assessment and collection of outcome, baseline, and other trial data, including any related processes to promote data quality (eg, duplicate measurements, training of assessors) and a description of study instruments (eg, questionnaires, laboratory tests) along with their reliability and validity, if known. Reference to where data collection forms can be found, if not in the protocol	Page 9-10
	18b	Plans to promote participant retention and complete follow-up, including list of any outcome data to be collected for participants who discontinue or deviate from intervention protocols	Page 11
Data management	19	Plans for data entry, coding, security, and storage, including any related processes to promote data quality (eg, double data entry; range checks for data values). Reference to where details of data management procedures can be found, if not in the protocol	Page 8
Statistical methods	20a	Statistical methods for analysing primary and secondary outcomes. Reference to where other details of the statistical analysis plan can be found, if not in the protocol	Page 11-12
	20b	Methods for any additional analyses (eg, subgroup and adjusted analyses)	Page 11
	20c	Definition of analysis population relating to protocol non-adherence (eg, as randomised analysis), and any statistical methods to handle missing data (eg, multiple imputation)	Page 11
**Methods: Monitoring**			
Data monitoring	21a	Composition of data monitoring committee (DMC); summary of its role and reporting structure; statement of whether it is independent from the sponsor and competing interests; and reference to where further details about its charter can be found, if not in the protocol. Alternatively, an explanation of why a DMC is not needed	n/a

For peer review only

	21b	Description of any interim analyses and stopping guidelines, including who will have access to these interim results and make the final decision to terminate the trial	n/a
Harms	22	Plans for collecting, assessing, reporting, and managing solicited and spontaneously reported adverse events and other unintended effects of trial interventions or trial conduct	Page 13
Auditing	23	Frequency and procedures for auditing trial conduct, if any, and whether the process will be independent from investigators and the sponsor	n/a
**Ethics and dissemination**			
Research ethics approval	24	Plans for seeking research ethics committee/institutional review board (REC/IRB) approval	Page 13
Protocol amendments	25	Plans for communicating important protocol modifications (eg, changes to eligibility criteria, outcomes, analyses) to relevant parties (eg, investigators, REC/IRBs, trial participants, trial registries, journals, regulators)	Page 13
Consent or assent	26a	Who will obtain informed consent or assent from potential trial participants or authorised surrogates, and how (see Item 32)	Page 5 and 13
	26b	Additional consent provisions for collection and use of participant data and biological specimens in ancillary studies, if applicable	n/a
Confidentiality	27	How personal information about potential and enrolled participants will be collected, shared, and maintained in order to protect confidentiality before, during, and after the trial	Page 8
Declaration of interests	28	Financial and other competing interests for principal investigators for the overall trial and each study site	16
Access to data	29	Statement of who will have access to the final trial dataset, and disclosure of contractual agreements that limit such access for investigators	Page 13
Ancillary and post-trial care	30	Provisions, if any, for ancillary and post-trial care, and for compensation to those who suffer harm from trial participation	n/a

For peer review only

Dissemination policy	31a	Plans for investigators and sponsor to communicate trial results to participants, healthcare professionals, the public, and other relevant groups (eg, via publication, reporting in results databases, or other data sharing arrangements), including any publication restrictions	Page 8 and 13
	31b	Authorship eligibility guidelines and any intended use of professional writers	Page 13
	31c	Plans, if any, for granting public access to the full protocol, participant-level dataset, and statistical code	n/a
**Appendices**			
Informed consent materials	32	Model consent form and other related documentation given to participants and authorised surrogates	n/a
Biological specimens	33	Plans for collection, laboratory evaluation, and storage of biological specimens for genetic or molecular analysis in the current trial and for future use in ancillary studies, if applicable	n/a

*It is strongly recommended that this checklist be read in conjunction with the SPIRIT 2013 Explanation & Elaboration for important clarification on the items. Amendments to the protocol should be tracked and dated. The SPIRIT checklist is copyrighted by the SPIRIT Group under the Creative Commons "Attribution-NonCommercial-NoDerivs 3.0 Unported" license.

For peer review only

For peer review only - http://bmjopen.bmj.com/site/about/guidelines.xhtml
